# Supplementary material for: Oxygen concentration modulates colibactin production
Source: Gut Microbes. 2023 Jun 13;15(1):2222437. doi: 10.1080/19490976.2023.2222437 (PMC10269391; doi:10.1080/19490976.2023.2222437)
Supplement: Supplemental Material [file KGMI_A_2222437_SM9989.docx]

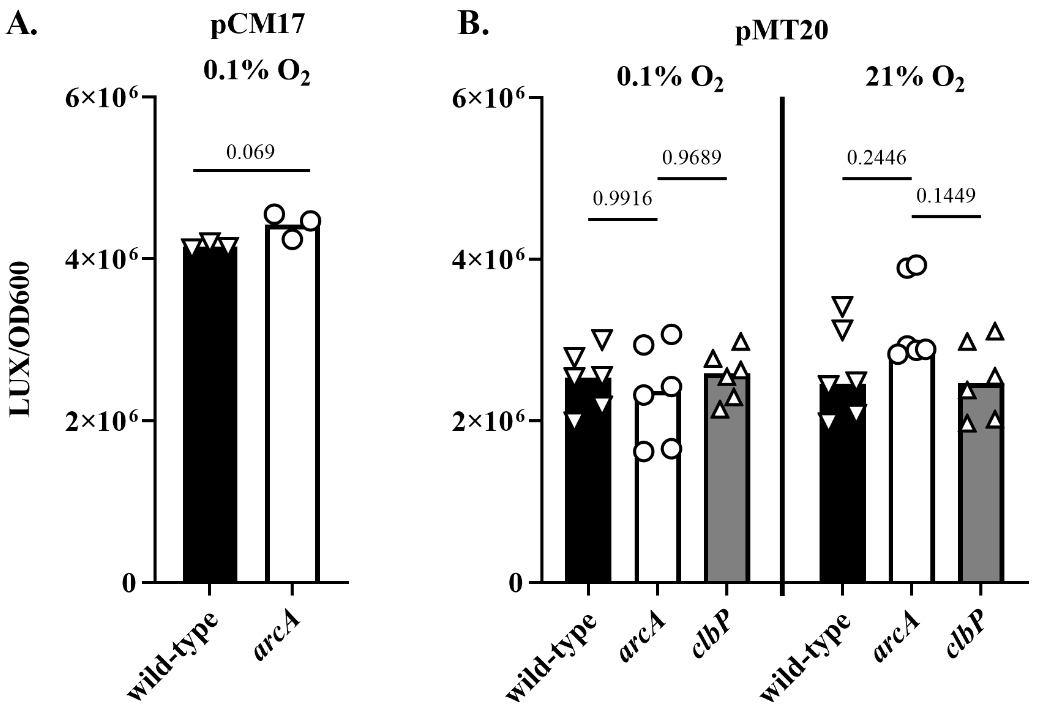
**Supplementary figure 1:**

**A.** Luminescence of *E. coli* strain SP15 carrying the luciferase reporter plasmid pCM17 (constitutive promoter upstream of *luxCDABE*) grown 3.5 h at 0.1% oxygen. The mean and individual values of three independent cultures are shown, with the p value of a t-test.

**B.** Luminescence of *E. coli* strain SP15 carrying the luciferase reporter plasmid pMT20 (promoter region of *clbS* upstream of *luxCDABE*) grown 3.5 h at 0.1% or 21% oxygen. The mean of 6 independent cultures are shown, with the p values of an ANOVA and Tukey's multiple comparison test.


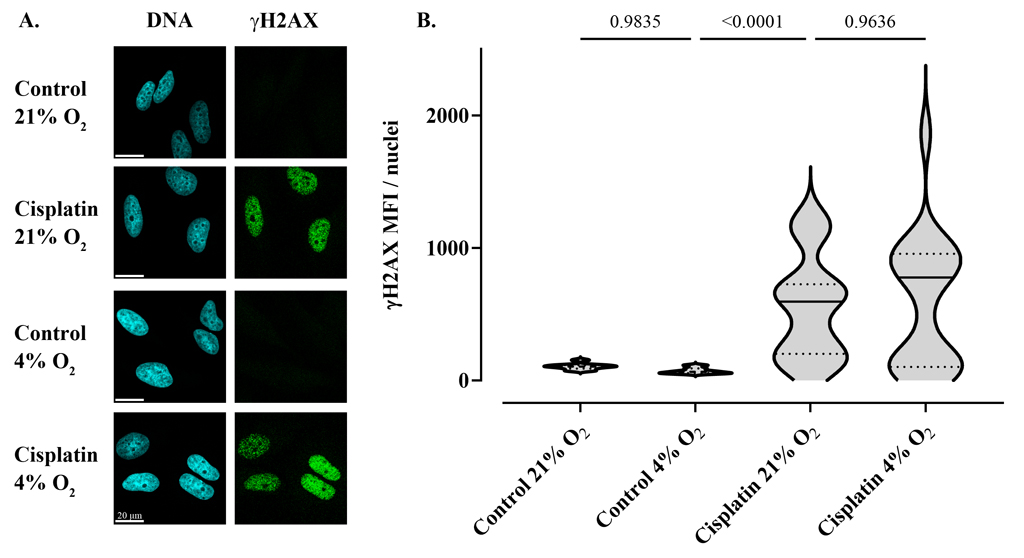


**Supplementary figure 2:** Oxygen does not alter the γH2AX response of genotoxin-treated HeLa cells.

**A.** HeLa epithelial cells were treated with cisplatin for 4 hours under 21% (“hyperoxic”) or 4% (“normoxic”) oxygen atmosphere, and then cellular DNA-damage was demonstrated by γH2AX immunofluorescent staining. DNA was counterstained with DAPI. Scale bar = 20 µm

**B.** γH2AX mean fluorescent intensity (MFI) within ~50 cell nuclei was measured by image analysis. The violin plots show the frequency distribution of the data, with the median and quartiles as solid and dotted lines. P values were calculated with an ANOVA and Tukey's multiple comparison test.


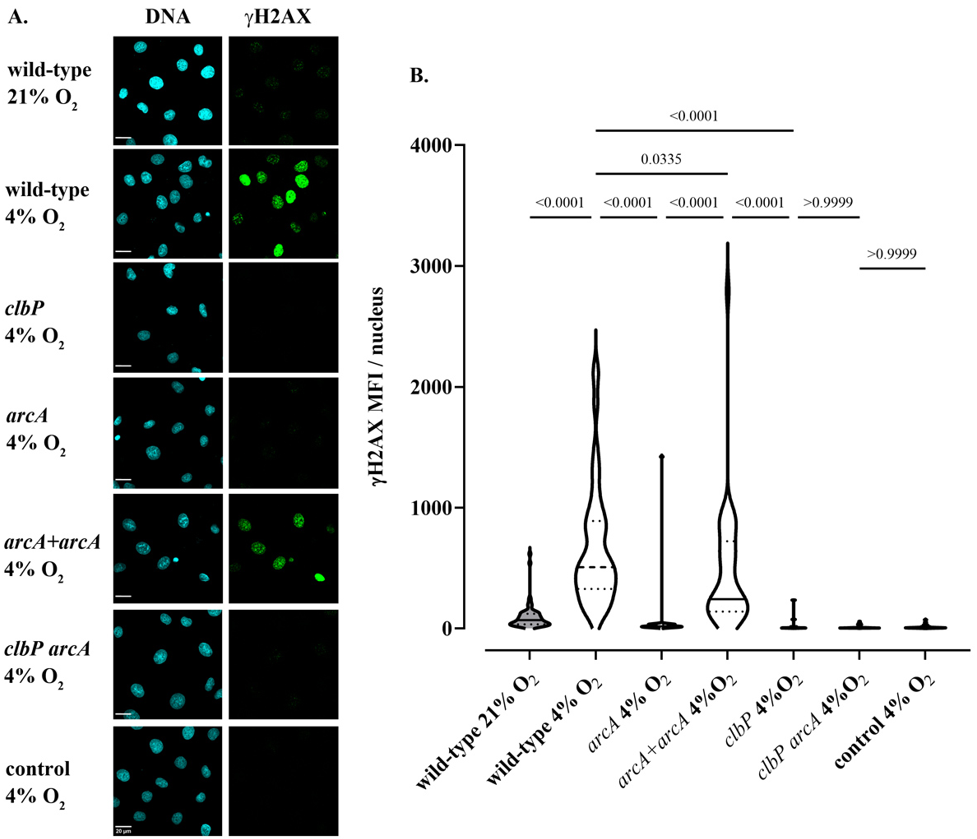
**Supplementary figure 3:** High oxygen concentration and *arcA* deletion inhibit the genotoxicity of colibactin-producing *E. coli* in intestinal IEC-6 cells.

**A.** Non-transformed rat intestinal epithelial IEC-6 cells were infected with *E. coli* wild-type strain SP15, the *clbP* mutant, the *arcA* mutant, the *arcA* mutant complemented with plasmid-encoded *arcA,* or the *clbP arcA* double mutant for 4 hours under 21% or 4% O_2_ atmosphere and then cellular DNA-damage was detected by γH2AX immunofluorescence staining. DNA was counterstained with DAPI. Scale bar = 20 µm

**B.** γH2AX mean fluorescence intensity (MFI) within approximatively 60 nuclei was measured by image analysis. The violin plots show the frequency distribution of the data, with the median and quartiles as solid and dotted lines. P values were calculated with an ANOVA and Tukey's multiple comparison test.


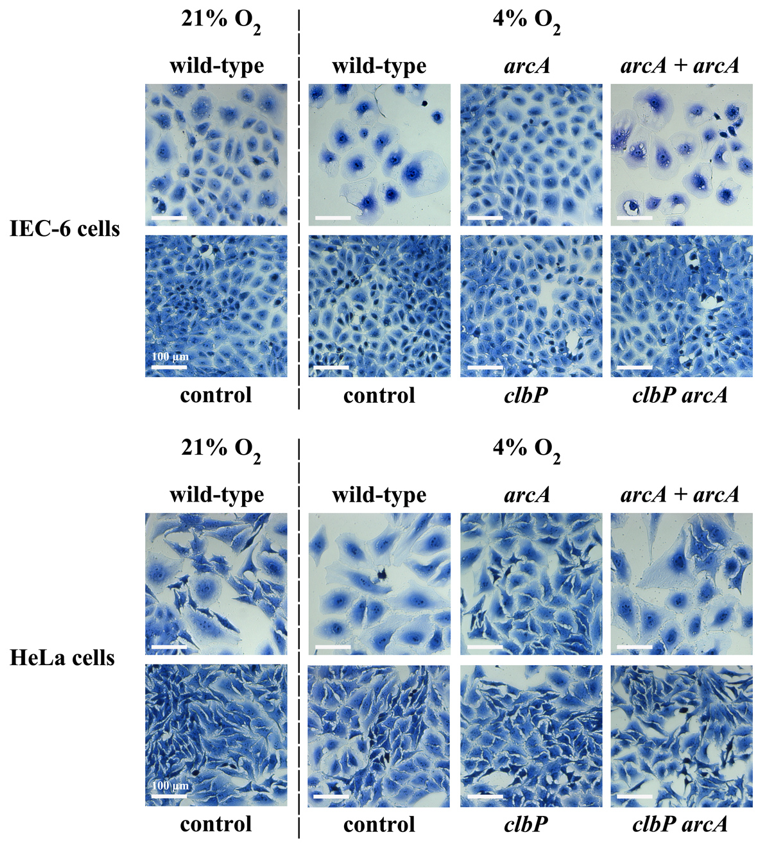
**Supplementary figure 4:** Cytotoxicity phenotype observed in IEC-6 and HeLa cells 48 h after infection with *E. coli* wild-type strain SP15, the *arcA* mutant, the *arcA* mutant complemented with plasmid-encoded *arcA,* the *clbP* mutant or the *clbP arcA* double mutant, under 21% or 4% O_2_ atmosphere. Control cells were left non-infected. Scale bars = 100 µm.
